# Differential regulation of specific genes in MCF-7 and the ICI 182780-resistant cell line MCF-7/182^R^-6

**DOI:** 10.1038/sj.bjc.6690061

**Published:** 1999-02

**Authors:** B L Jensen, J Skouv, B K Lundholt, A E Lykkesfeldt

**Affiliations:** Department of Tumor Endocrinology, Institute of Cancer Biology, Strandboulevarden 49, Copenhagen Ø, DK-2100, Denmark

**Keywords:** breast cancer, anti-oestrogen resistance, oestrogen receptor, progesterone receptor, gene expression

## Abstract

To elucidate the mechanisms involved in anti-oestrogen resistance, two human breast cancer cell lines MCF-7 and the ICI 182780-resistant cell line, MCF-7/182^R^-6, have been compared with regard to oestrogen receptor (ER) expression, ER function, ER regulation, growth requirements and differentially expressed gene products. MCF-7/182^R^-6 cells express a reduced level of ER protein. The ER protein is functional with respect to binding of oestradiol and the anti-oestrogens tamoxifen, 4-hydroxy-tamoxifen and ICI 182780, whereas expression and oestrogen induction of the progesterone receptor is lost in MCF-7/182^R^-6 cells. The ER protein and the ER mRNA are regulated similarly in the two cell lines when subjected to treatment with oestradiol or ICI 182780. Oestradiol down-regulates ER mRNA and ER protein expression. ICI 182780 has no initial effect on ER mRNA expression whereas the ER protein level decreases rapidly in cells treated with ICI 182780, indicating a severely decreased stability of the ER protein when bound to ICI 182780. In vitro growth experiments revealed that the ICI 182780-resistant cell line had evolved to an oestradiol-independent phenotype, able to grow with close to maximal growth rate both in the absence of oestradiol and in the presence of ICI 182780. Comparison of gene expression between the two cell lines revealed relatively few differences, indicating that a limited number of changes is involved in the development of anti-oestrogen resistance. Identification of the differentially expressed gene products are currently in progress. © 1999 Cancer Research Campaign


*British Journal of Cancer* (1999) **79**(3/4), 386–392
© 1999 Cancer Research Campaign
Article no. bjoc.1998.0061

### Differential regulation of specific genes in MCF-7 and the ICI 182780-resistant cell line MCF-7/182$^R$-6

**BL Jensen, J Skouv, BK Lundholt and AE Lykkesfeldt**

Department of Tumor Endocrinology, Institute of Cancer Biology, Danish Cancer Society, Strandboulevarden 49, DK-2100 Copenhagen Ø, Denmark

**Summary** To elucidate the mechanisms involved in anti-oestrogen resistance, two human breast cancer cell lines MCF-7 and the ICI 182780-resistant cell line, MCF-7/182$^R$-6, have been compared with regard to oestrogen receptor (ER) expression, ER function, ER regulation, growth requirements and differentially expressed gene products. MCF-7/182$^R$-6 cells express a reduced level of ER protein. The ER protein is functional with respect to binding of oestradiol and the anti-oestrogens tamoxifen, 4-hydroxy-tamoxifen and ICI 182780, whereas expression and oestrogen induction of the progesterone receptor is lost in MCF-7/182$^R$-6 cells. The ER protein and the ER mRNA are regulated similarly in the two cell lines when subjected to treatment with oestradiol or ICI 182780. Oestradiol down-regulates ER mRNA and ER protein expression. ICI 182780 has no initial effect on ER mRNA expression whereas the ER protein level decreases rapidly in cells treated with ICI 182780, indicating a severely decreased stability of the ER protein when bound to ICI 182780. In vitro growth experiments revealed that the ICI 182780-resistant cell line had evolved to an oestradiol-independent phenotype, able to grow with close to maximal growth rate both in the absence of oestradiol and in the presence of ICI 182780. Comparison of gene expression between the two cell lines revealed relatively few differences, indicating that a limited number of changes is involved in the development of anti-oestrogen resistance. Identification of the differentially expressed gene products are currently in progress.

**Keywords:** breast cancer; anti-oestrogen resistance; oestrogen receptor; progesterone receptor; gene expression

Many breast cancer patients with oestrogen receptor-positive primary tumours benefit from treatment with the anti-oestrogen tamoxifen (Mouridsen et al, 1978; Osborne et al, 1980). However, upon prolonged treatment with tamoxifen, resistant tumours will eventually develop. Recently, novel pure antagonistic compounds have been produced, to replace or supplement tamoxifen as first- or second-line endocrine treatment for breast cancer patients (Wakeling, 1993; Osborne et al, 1995). One of these agents, the pure steroidal anti-oestrogen ICI 182780, has been found to be a potent growth inhibitor of oestrogen-sensitive tissues in vivo and oestrogen-sensitive cell lines in vitro (Wakeling et al, 1991; Lykkesfeldt et al, 1994). Moreover, the ICI 182780 compound is effective for treatment of breast cancer patients refractory to tamoxifen therapy (Howell et al, 1995). Although initially very effective, treatment with the pure steroidal anti-oestrogen may postpone but not circumvent development of anti-oestrogen resistance (Lykkesfeldt et al, 1995; Osborne et al, 1995). Several different mechanisms have been proposed to account for anti-oestrogen resistance (Katzenellenbogen, 1991; Lykkesfeldt, 1996), and clinical evidence support some of them. In this study, we have compared the ICI 182780-resistant MCF-7 subline, MCF-7/182$^R$-6, with the parental ICI 182780-sensitive MCF-7 cell line with respect to the following parameters: ER content and binding characteristics, oestradiol and ICI 182780 regulation of ER and progesterone receptor (PgR) mRNA and protein, oestradiol requirement for in vitro cell growth, and gene expression in the presence of ICI 182780 to elucidate the mechanisms involved in anti-oestrogen resistance in this cell line.

*Received 5 March 1998*
*Revised 7 July 1998*
*Accepted 13 July 1998*

*Correspondence to:* AE Lykkesfeldt

### MATERIAL AND METHODS

### Cell lines and growth experiments

The MCF-7 cell line has been obtained from the Breast Cancer Task Force Cell Culture Bank, Mason Research Institute (Worchester, MA, USA). It has been adapted to grow in DME/F12 medium supplemented with 1% heat inactivated fetal calf serum (FCS) (Gibco, Life Technologies), 6 ng ml$^{-1}$ bovine insulin (Novo-Nordic, Copenhagen, Denmark) and 2.5 mM Glutamax (Life Technologies) (Briand and Lykkesfeldt, 1984). The MCF-7/182$^R$-6 cell line, which is resistant to the pure anti-oestrogen ICI 182780 (Zeneca Pharmaceuticals, Macclesfield, UK), has been established in this laboratory by long-term treatment of MCF-7 cells with $10^{-7}$ M ICI 182780, and it is maintained in DME/F12 medium supplemented with 1% FCS, 6 ng ml$^{-1}$ insulin, 2.5 mM Glutamax and $10^{-7}$ M ICI 182780 (Lykkesfeldt et al, 1995). Growth medium was changed every 2 or 3 days, and the cultures were subcultivated by trypsinization once a week (split ratio about 20).

MCF-7/182$^R$-6 cells were withdrawn from ICI 182780 1 week before onset of a growth experiment. MCF-7 and MCF-7/182$^R$-6 cells used for growth experiments were seeded with $2{\times}10^4$ cells per well in 24-well multidishes (Nunc, Roskilde, Denmark) in medium with 5% FCS from which steroids have been removed by a charcoal treatment (Horwitz and McGuire, 1978). Experimental medium was added after 2 days (day 0) and cell number determined at the indicated days. Experimental medium was renewed in the remaining wells after each cell counting.

### Competition assay and Scatchard analysis

Cytosols of MCF-7 and MCF-7/182R-6 cells were prepared as previously described (Briand and Lykkesfeldt, 1984). Cytosols were incubated at 4°C for 18–20 h in the presence of 1.5 nM [$^3$H] 17-β-oestradiol (Amersham, Buckinghamshire, UK), and different concentrations of (E)-*cis*-4-hydroxy-tamoxifen (Research Biochemicals International, Natick, MA, USA), tamoxifen, ICI 182780 (Zeneca), or oestradiol (Collaborative Research) in 96-well microtitre plates (Nunc). Unbound hormone was removed using dextran-coated charcoal (DCC). The results were corrected for non-specific binding by subtraction of the counts bound in a 100-fold excess of non-radioactive oestradiol, and expressed as a percentage of the maximal binding with [$^3$H]oestradiol alone. The molar concentrations leading to 50% competition ($IC_{50}$) were determined from competition curves. The equilibrium dissociation constant ($K_D$) was determined by the charcoal technique/Scatchard plot procedure (EORTC Breast Co-operative Group, 1980).

### Western analysis

MCF-7 and MCF-7/182R-6 cells were propagated in T25 flasks (Nunc) in ICI 182780-free medium 12 days before Western analysis to avoid any reminiscent effects. After treatment with either $10^{-9}$ M oestradiol or $10^{-7}$ M ICI 182780, the 70–80% confluent monolayer cultures were harvested using 0.5 ml RIPA buffer (100 mM sodium chloride, 20 mM Trizma-Base, 1% Triton X-100, 0.5% sodium desoxycholate, 0.1% SDS, and 1 mM sodium-EDTA, pH 8.0). Seventy micrograms of total protein per sample (determined by the Bio-Rad protein assay kit, Munich, Germany) were run on 15% sodium dodecyl sulphate polyacrylamide gel electrophoresis (SDS-PAGE) gels. The proteins were transferred to an Immobilon-P membrane (Millipore, Bedford, MA, USA) by electroblotting. Immunostaining was carried out using a primary monoclonal mouse anti-human ER antibody (1D5, DAKO, Glostrup, Denmark) and a secondary polyclonal rabbit anti-mouse IgG peroxidase conjugated antibody (P260, DAKO). The enhanced chemiluminescence (ECL) detection system (Amersham) was used for visualization of the ER protein as described in the manufacturer's manual.

### Northern analysis

MCF-7 and MCF-7/182R-6 cell cultures were treated with either $10^{-9}$ M oestradiol or $10^{-7}$ M ICI 182780 for 0, 1/4, 1/2, 1, 3, 6, 24 or 48 h before harvest with Trizol Reagent (Life Technologies). Total RNA was isolated from the Trizol Reagent lysate as recommended by the supplier. Poly(A)$^+$RNA was isolated with the Oligo $(dT)_{25}$ coupled magnetic beads (Dynal, Oslo, Norway) according to the manufacturer's manual. Two micrograms of poly(A)$^+$RNA were denatured with glyoxal/DMSO solution, run on a 1.2% agarose gel and transferred to a nylon membrane (Nytran 13N, Schleicher & Schuell, Dassel, Germany). The probes used for Northern hybridization were cDNA probes of the 1.8-kb *Eco*RI fragment of the hER from pSG5-HEGO (Tora et al, 1989), the 3.6-kb *Bam*HI fragment from the hPgR, and the 1.1-kb *Pst*I fragment of 36B4 from MCF-7 cloned into the pBR322 vector (Laborda, 1991) respectively. The probe for the differentially expressed transcript indicated by the arrow in Figure 5 was obtained by reamplification (using the same primer set and PCR conditions as for the screening) of DNA extracted from the area in the gel corresponding to the indicated band on the autoradiograph. The probes were randomly labelled with [α-$^{32}$P]dCTP (Amersham) using the Megaprime DNA labelling system (Amersham). Northern blots were hybridized using $1.25 \times 10^6$ c.p.m. randomly labelled probe ml$^{-1}$ QuickHyb Hybridization Solution (Stratagene). The blots were exposed to Kodak X-Omat AR-5 films. Each blot was also exposed to a PhosphorImager screen (Molecular Dynamics), for 1–4 days, before quantification using the ImageQuant software (Molecular Dynamics).

**Table 1** Molar concentration of oestradiol and anti-oestrogen leading to 50% competition ($IC_{50}$) with $^3$H-labelled 17-β-oestradiol for binding to ER in cytosol preparations from MCF-7 and MCF-7/182R-6 cells

| Hormone | MCF-7 (M) | MCF-7/182R-6 (M) |
|---|---|---|
| 17-β-oestradiol | $2.1 \times 10^{-9}$ | $1.7 \times 10^{-9}$ |
| ICI 182780 | $1.5 \times 10^{-7}$ | $1.0 \times 10^{-7}$ |
| (E)-*cis*-4-Hydroxy-tamoxifen | $3.6 \times 10^{-7}$ | $3.3 \times 10^{-7}$ |
| Tamoxifen | $1.2 \times 10^{-6}$ | $1.1 \times 10^{-6}$ |

### Differential display screening

RNA used for reverse transcriptase polymerase chain reaction (RT-PCR) differential display screening (Liang and Pardee, 1992) was isolated from MCF-7 and MCF-7/182R-6 cells using Trizol Reagent. Cells were grown either in absence or presence of $10^{-7}$ M ICI 182780 for 3 days before preparation. The RNA was treated with DNAase I (Promega) to remove possible contamination with DNA. The differential display screening was performed as recommended by the producer of the RNA Image kit (GenHunter, Nashville, TN, USA), but using [$^{33}$P]dATP instead of [$^{35}$S]dATP. cDNA was prepared from the DNAase-treated RNA using one of three downstream primers [$(T)_{11}$-G, -A, or -C, supplied in the kit] and the Moloney Murine Leukemia Virus Reverse Transcriptase (MMLV-RT, Gibco). The cDNA product was amplified for 40 cycles using one of eight arbitrary upstream primers (supplied in the kit) and the AmpliTaq DNA Polymerase (Perkin-Elmer). The RT and the PCR reactions were performed in a Perkin-Elmer 9600 thermocycler. The RT-PCR products were run on a 5% denaturing polyacrylamide gel. All reactions were run in duplicate and all 24 primer combinations were tested. After electrophoresis, the gel was vacuum dried and placed in a film cassette with intensifying screen for autoradiography using Kodak X-Omat AR-5 films. Reamplified, gel-purified DNA fragments were used as templates for solid-phase DNA sequencing as described previously (Madsen et al, 1995).

### RESULTS

### Expression and binding characteristics of the ER protein in MCF-7 and MCF-7/182R-6 cells

Previous work from our group has documented that loss of ER is not involved in the emergence of the ICI 182780-resistant phenotype in our cell lines established in vitro (Lykkesfeldt et al, 1995). However, the ER content in the resistant MCF-7/182R-6 cells is only 221 ± 58 fmol mg$^{-1}$ cytosol protein compared with 616 ± 93 fmol mg$^{-1}$ cytosol protein in the parent MCF-7 cells, demonstrating a reduced expression of the ER protein in the resistant cell line (Larsen et al, 1997). Whether or not the ER protein

© Cancer Research Campaign 1999

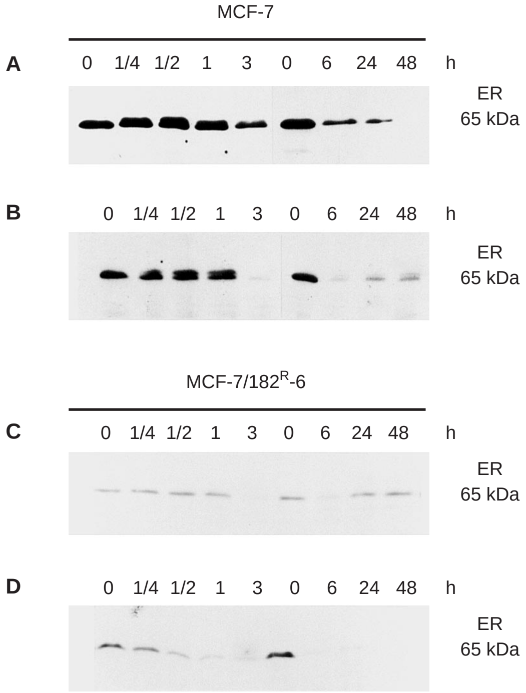

**Figure 1** Effect of oestradiol or ICI 182780 on ER protein content in MCF-7 and MCF-7/182R-6 cells. Cell lysates were prepared at the indicated treatment times with either $10^{-9}$ M oestradiol (**A** and **C**) or $10^{-7}$ M ICI 182780 (**B** and **D**). Seventy micrograms of protein were loaded in each lane on a 15% SDS-PAGE gel. The ER protein was detected using a primary monoclonal mouse anti-human ER antibody (1D5), a secondary polyclonal rabbit anti-mouse IgG peroxidase conjugated antibody (P260), and the immunocomplex was visualized using the enhanced chemiluminescence (ECL) detection system (Amersham)

expressed in MCF-7/182R-6 cells is functional primarily depends on its ability to bind the physiological ligand oestradiol. Scatchard plot analysis of results from a ligand binding assay with oestradiol revealed very similar $K_D$ values of $0.65 \times 10^{-10}$ M and $1.34 \times 10^{-10}$ M for MCF-7 and MCF-7/182R-6 respectively. The possibility that the oestrogen receptors, expressed in the MCF-7/182R-6 cell line, were unable to bind ICI 182780 or other anti-oestrogens like 4-hydroxy-tamoxifen and tamoxifen was investigated using a competition assay. It was clearly demonstrated that both oestradiol and anti-hormones were competing with $^3$H-labelled oestradiol for binding to the ER, each at identical molar concentrations in both cell lines (Table 1).

### Effect of oestradiol or ICI 182780 on the ER protein expression

To determine how the ER protein is affected by oestradiol and ICI 182780, time course Western blot analyses were carried out. In both MCF-7 and MCF-7/182R-6 cells, only one ER species was present, corresponding to the 65-kDa wild-type ER protein (Figure 1). The ER protein level decreased significantly in the cells after a few hours of treatment with either $10^{-9}$ M oestradiol or $10^{-7}$ M ICI 182780 (Figure 1). In MCF-7 cells, total disappearance of the ER protein was found upon 48 h of treatment with oestradiol, or upon 3 h of treatment with ICI 182780. A very slight increase in ER level was seen after 24 and 48 h of treatment with ICI 182780. In MCF-7/182R-6 cells, both oestradiol and ICI 182780 rapidly down-regulated the ER protein content to a minimum after 3–6 h. However, unlike MCF-7 cells, reappearance of ER protein after 24 and 48 h of ICI 182780 treatment was not observed in MCF-7/182R-6 cells. The level of ER protein in MCF-7/182R-6 cells is

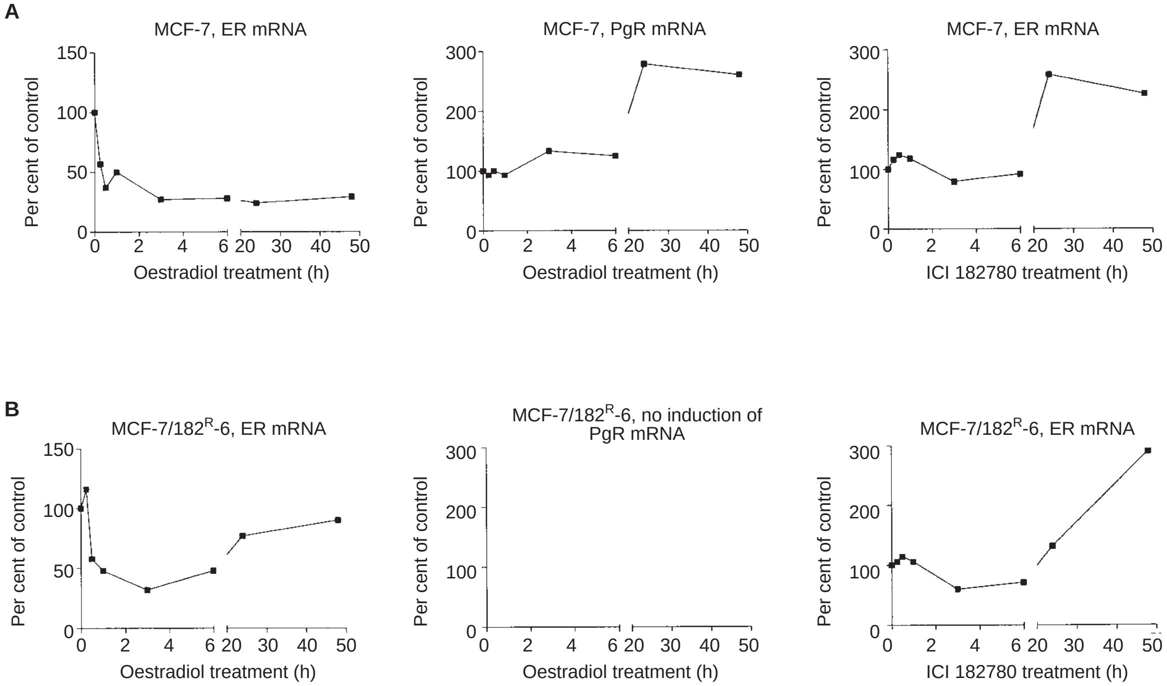

**Figure 2** Effect of $10^{-9}$ M oestradiol on the ER mRNA and PgR mRNA expression and of $10^{-7}$ M ICI 182780 on the ER mRNA expression in MCF-7 (**A**) and MCF-7/182R-6 cells (**B**). RNA was prepared from monolayer cell cultures after 0, 1/4, 1/2, 1, 3, 6, 24, and 48 h of treatment with either oestradiol or ICI 182780. The RNA was run on an agarose gel, blotted onto a nylon membrane, before the amount of transcripts of ER, PgR and 36B4 were determined by hybridization with randomly labelled plasmid probes. The 36B4 mRNA was used as an internal control and the PhosphorImager scan values were normalized to this transcript. The ratio of either ER or PgR and 36B4 was expressed as a percentage of the control ratio obtained in RNA samples from non-steroid-treated cells

 © Cancer Research Campaign 1999

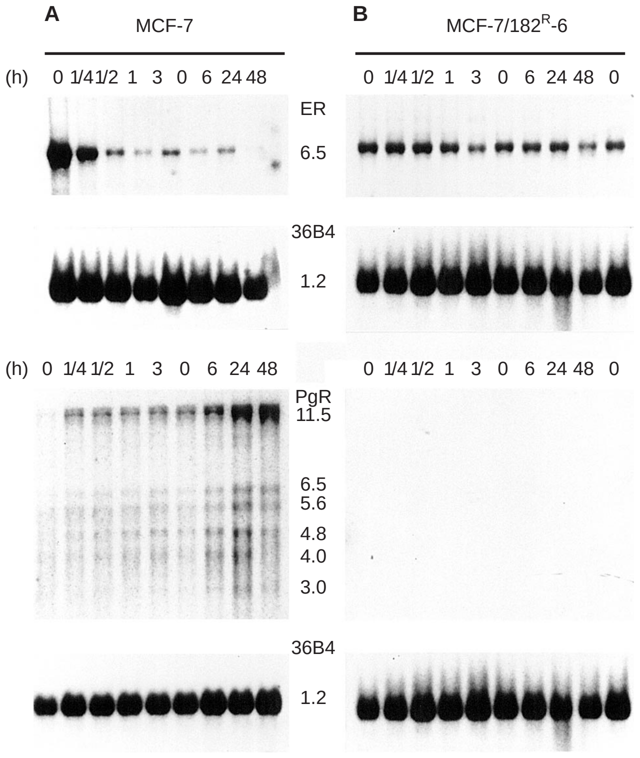

**Figure 3** Northern blots of RNA from MCF-7 (**A**) and MCF-7/182R-6 cells (**B**) treated with oestradiol. Two micrograms of poly(A)+RNA from each cell line were analysed by Northern hybridization. 32P-labelled plasmid probes for ER and PgR were used, and the blots were rehybridized with 36B4 probe as internal control. The sizes of the individual transcripts are shown, the ER mRNA is 6.5 kb, the PgR-A mRNAs are 5.6 and 3.0 kb, the PgR-B mRNAs are 11.5, 6.5, 4.8 and 4.0 kb, and the 36B4 mRNA is 1.2 kb

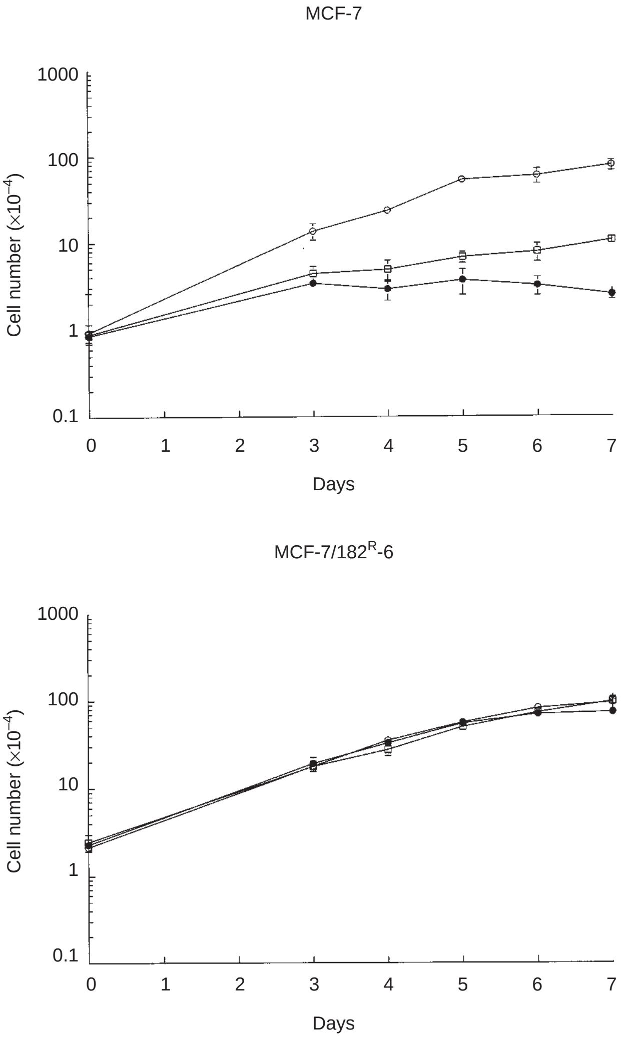

**Figure 4** For the growth experiments, MCF-7 or MCF-7/182R-6 cells were seeded in multi-dishes ($2\times10^4$ cells per well). Two days after seeding, the medium containing 5% oestradiol-deprived FCS was changed to experimental medium supplemented either with: no steroid (□); $10^{-9}$ M oestradiol (○); or $10^{-7}$ M ICI 182780 (●). At day 0, 3, 4, 5, 6 and 7 after the addition of the experimental medium, the cells in four wells from each culture were counted in a Bürker-Türck chamber, and cell numbers were expressed as a function of time. Standard deviations are indicated with vertical bars significantly lower than in MCF-7 cells, and a small increase may escape detection by this method. Cells followed upon removal of ICI 182780 from the growth medium exhibited no reappearance of ER protein within 48 h (data not shown), suggesting that longer growth in ICI 182780-free medium is required to re-establish a normal ER level.

### Effect of oestradiol or ICI 182780 on the ER and PgR mRNA expression

To elucidate whether the effect of oestradiol and ICI 182780 on the ER protein were exerted at the transcriptional or the translational level, and to examine the possible induction of the PgR mRNA synthesis, time course Northern blotting experiments were carried out. In Figure 2, graphical representations of data from PhosphorImager scanning of Northern blots (Figure 3), expressed as the ratios between the specific transcript and the 36B4 ribosomal phosphoprotein, are shown. The mRNA levels (in percentage of control RNA from non-steroid-treated cells) are expressed as a function of time. In MCF-7 cells, ER mRNA was down-regulated by $10^{-9}$ M oestradiol to 30% after about 30 min, and the expression remained low throughout the entire time interval of 48 h. In contrast, in the resistant cell line, the ER mRNA was down-regulated to 50% within the first few hours, but tended to increase after 24 and 48 h. The PgR expression was increased approximately threefold after 24 h of oestradiol treatment in the parental cell line. However, the resistant cell line had totally lost the ability to express, and to induce the expression of the PgR mRNA upon oestradiol treatment, levels above the detection level for Northern blotting. The ER mRNA expression was unaffected by the addition of $10^{-7}$ M ICI 182780 until after 24 and 48 h, when an up-regulation to 240% and 270% of the control level was seen in MCF-7 and MCF-7/182R-6 cells respectively. Cultures of both cell lines treated with ICI 182780 even for short time intervals (of less than 1 h) did not express measurable amounts of PgR mRNA (data not shown). The effects described above were found in three individual experiments for each of the two cell lines. Photos of representative Northern blots, with the characteristic absence of PgR induction in the resistant cell line upon oestradiol treatment, are shown in Figure 3.

© Cancer Research Campaign 1999

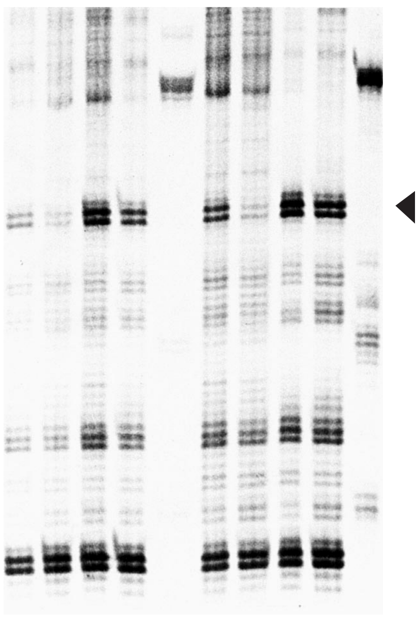

Figure 5 Autoradiogram showing expressed gene products in MCF-7 and MCF-7/182R-6. Genes expressed in MCF-7 cells are shown in lanes 1 and 2, and genes expressed in MCF-7/182R-6 cells are shown in lanes 3 and 4. The samples run in lanes 1 and 3 originate from cells maintained in control medium (1% FCS), whereas the samples run in lanes 2 and 4 are from cells treated with ICI 182780 for 3 days before RNA preparation and differential display. C indicates control RNA from rat embryo fibroblasts supplied in the differential display kit from GenHunter. The primer set used for the duplicate reactions was upstream primer number 3 and downstream primer H-$T_{11}$-C

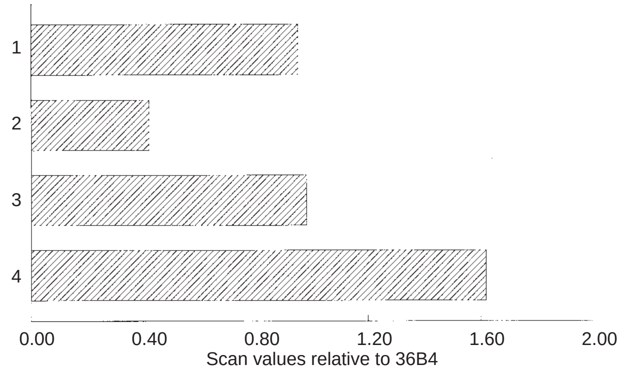

**Figure 6** Relative content of a differentially expressed specific mRNA determined by Northern analysis and PhosphorImager quantification. Northern blot analysis of the differentially expressed transcript, identified from the differential display gel shown in Figure 5. The numbers 1–4 correspond to RNA samples obtained from MCF-7 (1 and 2) and MCF-7/182R-6 (3 and 4) cells grown either in absence (1 and 3) or presence (2 and 4) of $10^{-7}$ M ICI 182780 for 3 days before RNA preparation. The PhosphorImager quantification of the differentially expressed transcripts is related to the internal control transcript, 36B4

### Growth of MCF-7 and MCF-7/182R-6 cells in oestradiol-deprived medium

The precise mechanism(s) behind the capacity of MCF-7/182R-6 cells to tolerate ICI 182780 well is not known at present. To investigate whether the MCF-7/182R-6 cell line has evolved to an oestradiol-independent or an oestradiol-hypersensitive phenotype, growth experiments in medium containing 5% FCS, stripped for oestrogen compounds, were conducted (Figure 4). MCF-7 cells grow slowly in oestradiol-deprived medium, whereas MCF-7 cultures supplied with $10^{-9}$ M oestradiol grow rapidly with a cell population doubling time of about 20 h. MCF-7 cells treated with $10^{-7}$ M ICI 182780 display a small initial increase in cell number, but after 4 days of treatment a decrease in cell number, due to cell death, is observed. In contrast, the MCF-7/182R-6 cells grow equally well in the absence or the presence of oestradiol. Cell population doubling time is 24 h for the first 5 days. These results indicate that the anti-oestrogen-resistant cell line has lost the oestradiol requirement for growth in vitro. However, because oestrogen-deprived serum contains low levels of oestrone and oestrone sulphate (Briand and Lykkesfeldt, 1984), which may be converted to oestradiol, the ability of MCF-7/182R-6 cells to grow in oestradiol-deprived medium may either be due to a significantly reduced requirement for oestradiol (hypersensitivity) (Masamura et al, 1995) or to development of oestrogen-independent cell growth. To elucidate this, MCF-7/182R-6 cells were grown with $10^{-7}$ M ICI 182780. Such a high concentration of the pure anti-oestrogen should be more than sufficient to compete totally with any low amount of oestrogen, and consequently the observed rapid growth of MCF-7/182R-6 cells under these conditions (Figure 4) demonstrates that the resistant cell line is oestrogen-independent for in vitro cell growth.

### Differential display screening of cells grown in presence or absence of ICI 182780

To search for gene products which may be responsible for the development of anti-oestrogen resistance in vitro, differential display screening was initiated (Liang and Pardee, 1992). Control experiments confirming the reproducibility between different passages of cells were performed before the screening was carried out with only one passage of each cell line. Relatively few gene expression differences were identified as MCF-7 and MCF-7/182R-6 RNA from cultures grown in the presence or the absence of $10^{-7}$ M ICI 182780 for 3 days were compared. In total, we have performed 24 separate RT-PCR reactions and found 35 differences. Figure 5 shows a typical autoradiograph, in which each reaction has been run in duplicate, displaying gene expression in MCF-7 and MCF-7/182R-6 cells. The differentially expressed gene product indicated with an arrowhead has been isolated from the gel, reamplified and sequenced. Northern analysis showed that this specific gene product is down-regulated in MCF-7, whereas it is modestly up-regulated in MCF-7/182R-6 cells upon ICI 182780 treatment (Figure 6). It was found that the gene product, indicated with the arrowhead in Figure 5, is homologous to human mitochondrial DNA control region (access code J01415). The DNA sequence, corresponding to our mRNA, represents a so far unidentified gene product. Several other gene expression differences than the one displayed in Figure 5 are currently being investigated.

### DISCUSSION

A panel of anti-oestrogen-resistant cell lines have been developed in this laboratory upon long-term treatment of MCF-7 cells with different anti-oestrogens (Lykkesfeldt et al, 1995). The MCF-7 breast cancer cell line, which expresses high levels of ER, is responsive to mitogenic and growth inhibitory signals induced by

 © Cancer Research Campaign 1999

oestrogens and anti-oestrogens respectively. Therefore, MCF-7 and sublines hereof provide excellent model systems for the study of hormonal regulation of ER in human breast cancer. Most tumours responding to endocrine therapy with anti-oestrogens are ER positive. However, the stability of this phenotype may not be permanent (Johnston et al, 1995; Clarke and Brünner, 1996; Robertson, 1996). This fact prompted us to investigate whether loss of ER or severely changed regulation and function of the ER could explain the emergence of the ICI 182780-resistant phenotype of MCF-7/182$^R$-6 cells. Furthermore, we compared the parental MCF-7 cell line and the MCF-7/182$^R$-6 cell line with regard to growth requirements and differentially expressed gene products, and to gain more knowledge of which precise mechanism(s) might be involved in the development of anti-oestrogen resistance.

Quantitative ER determinations have shown that the parental cell line expresses approximately threefold more ER than MCF-7/182$^R$-6 cells (Larsen et al, 1997). The hormone/anti-hormone binding studies revealed similar $K_d$ values for oestradiol binding and identical $IC_{50}$ concentrations for competition of oestradiol with the anti-oestrogens ICI 182780, 4-hydroxy-tamoxifen and tamoxifen. Therefore, we conclude that the anti-oestrogen resistance is not caused by impaired receptor function with respect to binding of oestrogen or anti-oestrogen.

Western blot analyses disclosed that oestradiol as well as ICI 182780 were able to down-regulate the content of ER protein effectively in both cell lines, after only a few hours of treatment (Figure 1). These semiquantitative results are in accordance with the observed $t_{1/2}$ of 3 h and 0.9 h for the ER in the presence of oestradiol or ICI 164384, reported earlier (Dauvois et al, 1992; Borrás et al, 1996). The level of regulation of the ER expressed in our cell lines was determined by comparing the time course Western blots with corresponding Northern blots. It was found that in MCF-7 cells oestradiol down-regulated the ER mRNA to about 30% within the first hours of treatment, as reported by others (Saceda et al, 1988; Berkenstam et al, 1989; Berthois et al, 1990; Borrás et al, 1994). However, the initial reduction in the ER protein level in both cell lines more than corresponds with the reduction to 30–50% in the mRNA level, suggesting that oestradiol regulates ER protein content both by reducing the mRNA synthesis and also by destabilizing the protein, e.g. via ER processing as described by Horwitz and McGuire (1978). We have no explanation for the observation that MCF-7/182$^R$-6 cells appear to differ from MCF-7 cells with respect to re-establishment of control ER mRNA and protein level after oestradiol treatment for 24 and 48 h. Total disappearance of ER protein in both cell lines after 3 h of ICI 182780 treatment without reduction in ER mRNA level is in agreement with observations of others (Gibson et al, 1991; Dauvois et al, 1992; Pink and Jordan, 1996), indicating that the antagonistic effect of the pure anti-oestrogens ICI 164384 and ICI 182780 is due to ER protein loss. The fact that the ER, by which ICI 182780 exerts its function, has maintained the same pattern of regulation in the resistant cell line compared with the MCF-7 cell line, both regarding ER protein and ER mRNA, indicates that the resistant cell line, when growing with high ICI 182780 concentration, may compensate for the blocked ER pathway by inducing other mitogenic signals in the cells. The activation of such mitogenic pathways may account for the ability of MCF-7/182$^R$-6, opposed to MCF-7, to grow well in the presence of ICI 182780.

Analysis of PgR mRNA expression revealed significant differences between the two cell lines. Whereas the PgR mRNA expression was increased about threefold in the parental cell line upon 48 h of oestradiol treatment, no detectable transcripts were present in poly(A)$^+$RNA preparations from MCF-7/182$^R$-6 cells either before or after oestradiol treatment (Figure 3). These findings agree with previous reports both from this laboratory and others, showing that several anti-oestrogen-resistant cell lines lack PgR expression or express reduced levels of PgR (Lykkesfeldt et al, 1994, 1995; Herman and Katzenellenbogen, 1996; Pink and Jordan, 1996). It is very likely that a certain threshold level of ER is necessary for the induction and synthesis of ER-regulated gene products, such as the PgR (van Agthoven et al, 1994). The required level of ER for induction of transcription may vary from gene to gene, as shown for vascular endothelial growth factor and c-*fos* (Hyder et al, 1997) and supported by our observation that the cathepsin D and pS2 genes display a normal oestrogen response in anti-oestrogen-resistant cell lines (Larsen et al, 1997). Therefore, we presume that the reduced ER level in the MCF-7/182$^R$-6 cells is insufficient for PgR induction. However, other intracellular changes such as lack of cofactors essential for the ER-governed transcription of the PgR gene, or mutations in the promoter region of the PgR gene leading to decreased initiation of transcription, may also account for the lost PgR expression and inducibility in the MCF-7/182$^R$-6 cell line.

To investigate to which degree of oestrogen independence the MCF-7/182$^R$-6 cells have evolved, cell growth experiments were performed using serum from which endogenous oestrogens have been removed by a charcoal treatment. The resistant cell line exhibits a decreased requirement for oestradiol because the cell line is able to grow well in oestradiol-deprived medium. Similar results have been reported for a hydroxy-tamoxifen-resistant cell line (Herman and Katzenellenbogen, 1996), but the reason for the decreased oestradiol requirement remains to be determined. Oestrogen-deprived serum contains small amounts of oestrone and oestrone sulphate (Briand and Lykkesfeldt, 1984), and the MCF-7/182$^R$-6 cell line may be able to respond to low amounts of oestrogen as shown for a MCF-7 subline in which oestradiol deprivation caused hypersensitivity (Masamura et al, 1995). Our observation that MCF-7/182$^R$-6 cells are able to grow equally well in oestradiol-deprived medium with and without $10^{-7}$ M ICI 182780 eliminated the possibility of hypersensitivity to oestradiol because the pure anti-oestrogen is able to compete completely with low concentration of reminiscent oestrogen. The conclusion from our growth experiments is that the MCF-7/182$^R$-6 cells grow as oestrogen-independent cells in vitro. It should be mentioned that MCF-7/182$^R$-6 cells require oestrogen for growth in vivo in athymic nude mice (Lykkesfeldt et al, 1995; Larsen et al, 1997), although the appearance of slow-growing tumours in some of the intact animals without oestrogen supplementation indicates a reduced requirement for oestrogen (Lykkesfeldt et al, 1995).

Analysis of differentially expressed genes was performed to investigate the similarity of the two cell lines. In general, the gene expression is very much the same in the two cell lines, but 35 differentially expressed transcripts were displayed, as DNA-free RNA from MCF-7 and MCF-7/182$^R$-6 cells treated or non-treated with ICI 182780 for 3 days were compared. According to the manufacturer, our 24 RT-PCR reactions should display about 10% of all 15 000 genes assumed to be expressed in a cell. Our finding of 35 differences indicates that approximately 2.5% of the genes are differently regulated in MCF-7 and MCF-7/182$^R$-6 cells after 3 days of ICI 182780 treatment. The identity of the majority of these displayed differences still remains to be determined, but a few differentially expressed transcripts have been identified. One of the identified mRNAs represents a mitochondrial gene, which

© Cancer Research Campaign 1999

according to a DNA database search may encode a protein with a high level of homology to prokaryotic membrane lipoproteins. We believe that this gene product, rather than being the reason for the development of anti-oestrogen resistance, is the consequence of the altered expression of one or more so far unidentified gene product(s). The identity of such 'anti-oestrogen resistance-inducing gene products' is still unknown, but once identified they will provide us with valuable knowledge of the precise mechanism(s) by which anti-oestrogen resistance emerges and hopefully also supply us with some indication to what might be done to avoid or postpone the development of resistance in vivo.

In conclusion, our results demonstrate that the ICI 182780 resistant cell line, MCF-7/182$^R$-6, displays oestrogen-independent growth in vitro, lack of PgR expression and diffential expression of some genes. However, the cell line remains closely related to the parental MCF-7 cell line, with respect to expression, function and regulation of the ER protein and ER mRNA. These findings, together with similar findings by others (Herman and Katzenellenbogen, 1996; Larsen et al, 1997), lead us to suggest that the resistant cell line represents an early stage in the development of a totally hormonal and growth factor-independent phenotype.

### ACKNOWLEDGEMENTS

We would like to thank Dr P Chambon for providing the ER, PgR and 36B4 probes, and Mrs I Heiberg and Mrs B Reiter for excellent technical assistance. Tamoxifen and ICI 182780 were kindly provided by Zeneca Pharmaceuticals, Macclesfield, UK. Financial support from the Danish Cancer Society and Mrs Astrid Thaysen's Foundation is gratefully acknowledged.

### REFERENCES

Berkenstam A, Glaumann H, Martin M, Gustafsson JÅ and Norsted G (1989) Hormonal regulation of estrogen receptor messenger ribonucleic acid in T47D$_{co}$ and MCF-7 breast cancer cells. *Mol Endocrinol* **3**: 22–28

Berthois Y, Dong XF, Roux-Dossetto M and Martin PM (1990) Expression of estrogen receptor and its messenger ribonucleic acid in the MCF-7 cell line: multiparametric analysis of its processing and regulation by estradiol. *Mol Cell Endocrinol* **74**: 11–20

Borrás M, Hardy L, Lempereue F, El Khissiin AH, Legros N, Gol-Winkler R and Leclercq G (1994) Estrogen-induced down-regulation of estrogen receptor. Effect of various modulators of protein synthesis and expression. *J Steroid Biochem Mol Biol* **48**: 325–336

Borrás M, Laios I, El Khissiin A, Seo HS, Lempereur F, Legros N and Leclercq G (1996) Estrogenic and antiestrogenic regulation of the half-life of covalently labeled estrogen receptor in MCF-7 breast cancer cells. *J Steroid Biochem Mol Biol* **57**: 203–213

Briand P and Lykkesfeldt AE (1984) Effect of estrogen and antiestrogen on the human breast cancer cell line MCF-7 adapted to grow at low serum concentration. *Cancer Res* **44**: 1114–1119

Clarke R and Brünner N (1996) Acquired estrogen independence and antiestrogen resistance in breast cancer. Estrogen receptor driven phenotypes? *Trends Endocrinol Metab* **7**: 291–301

Dauvois S, Danielian PS, White R and Parker MG (1992) Antiestrogen ICI 164,384 reduces cellular estrogen receptor content by increasing its turnover. *Proc Natl Acad Sci USA* **89**: 4037–4041

EORTC Breast Co-operative Group (1980) Revision of the standards for the assessment of hormone receptors in human breast cancer; report of the second EORTC workshop, held on 16–17 March, 1979, in the Netherlands Cancer Institute. *Eur J Cancer* **16**: 1513–1515

Gibson MK, Nemmers LA, Beckman WC, Davis VL, Curtis SW and Korack KS (1991) The mechanism of ICI 164,384 antiestrogenicity involves rapid loss of ER in uterine tissue. *Endocrinology* **129**: 2000–2010

Herman ME and Katzenellenbogen BS (1996) Response-specific antiestrogen resistance in a newly characterized MCF-7 human breast cancer cell line resulting from long-term exposure to trans-hydroxytamoxifen. *J Steroid Biochem Mol Biol* **59**: 121–134

Horwitz KB and McGuire WL (1978) Nuclear mechanisms of estrogen action. *J Biol Chem* **253**: 8185–8191

Howell A, DeFriend DJ, Robertson JFR, Blamey RW and Walton P (1995) Response to a specific antioestrogen (ICI 182780) in tamoxifen-resistant breast cancer. *Lancet* **345**: 29–30

Hyder SM, Chiappetta C, Murthy L and Stancel GM (1997) Selective inhibition of estrogen-regulated gene expression in vivo by the pure antiestrogen ICI 182,780. *Cancer Res* **57**: 2547–2549

Johnston SRD, Saccani-Jotti G, Smith IE, Salter J, Newby J, Coppen M, Ebbs SR and Dowsett M (1995) Changes in estrogen receptor, progesterone receptor, and pS2 expression in tamoxifen-resistant human breast cancer. *Cancer Res* **55**: 3331–3338

Katzenellenbogen BS (1991) Antiestrogen resistance: mechanisms by which breast cancer cells undermine the effectiveness of endocrine therapy. *J Natl Cancer Inst* **83**: 1434–1444

Laborda J (1991) 36B4 cDNA used as an estradiol-independent mRNA control is the cDNA for human acidic ribosomal phosphoprotein PO. *Nucleic Acids Res* **19**: 3998

Larsen SS, Madsen MW, Jensen BL and Lykkesfeldt AE (1997) Resistance of human breast-cancer cells to the pure steroidal anti-estrogen ICI 182,780 is not associated with a general loss of estrogen-receptor expression or lack of estrogen responsiveness. *Int J Cancer* **72**: 1129–1136

Liang P and Pardee AB (1992) Differential display of eukaryotic messenger RNA by means of the polymerase chain reaction. *Science* **257**: 967–970

Lykkesfeldt AE (1996) Mechanisms of tamoxifen resistance in the treatment of advanced breast cancer. *Acta Oncol* **35**: 9–14

Lykkesfeldt AE, Madsen MW and Briand P (1994) Altered expression of estrogen-regulated genes in a tamoxifen-resistant and ICI 164,384 and ICI 182,780 sensitive human breast cancer cell line, MCF-7/TAM$^R$-1. *Cancer Res* **54**: 1587–1595

Lykkesfeldt AE, Larsen SS and Briand P (1995) Human breast cancer cell lines resistant to pure anti-estrogens are sensitive to tamoxifen treatment. *Int J Cancer* **61**: 529–534

Madsen MW, Reiter BE and Lykkesfeldt AE (1995) Differential expression of estrogen receptor mRNA splice variants in the tamoxifen resistant human breast cancer cell line, MCF-7/TAM$^R$/TAM$^R$-1 compared to the parental MCF-7 cell line. *Mol Cell Endocrinol* **109**: 197–207

Masamura S, Santner SJ, Heitjan DF and Santen RJ (1995) Estrogen deprivation causes estradiol hypersensitivity in human breast cancer cells. *J Clin Endocrinol Metab* **80**: 2918–2925

Mouridsen H, Palshof T, Patterson J and Battersby L (1978) Tamoxifen in advanced breast cancer. *Cancer Treat Rev* **5**: 131–141

Osborne CK, Yochmowitz MG, Knight WA and McGuire WL (1980) The value of estrogen and progesterone receptors in the treatment of breast cancer. *Cancer* **46**: 2884–2888

Osborne CK, Coronado-Heinsohn EB, Hilsenbeck SG, McCue BL, Wakeling AE, McClelland RA, Manning DL and Nicholson RI (1995) Comparison of the effects of pure steroidal antiestrogen with those of tamoxifen in a model of human breast cancer. *J Natl Cancer Inst* **87**: 746–750

Pink JJ and Jordan VC (1996) Models of estrogen receptor regulation by estrogens and antiestrogens in breast cancer cell lines. *Cancer Res* **56**: 2321–2330

Robertson JFR (1996) Oestrogen receptor: a stable phenotype in breast cancer. *Br J Cancer* **73**: 5–12

Saceda M, Lippman ME, Chambon P, Lindsey RL, Ponglikitmongkol M, Puente M and Martin MB (1988) Regulation of the estrogen receptor in MCF-7 cells by estradiol. *Mol Endocrinol* **2**: 1157–1162

Tora L, White J, Brou C, Tasset D, Webster N, Scheer E and Chambon P (1989) The human estrogen receptor has two independent nonacidic transcriptional activation functions. *Cell* **59**: 477–487

van Agthoven T, van Agthoven TLA, Dekker A, Foekens JA and Dorssers LCJ (1994) Induction of estrogen independence of ZR-75-1 human breast cancer cells by epigenetic alterations. *Mol Endocrinol* **8**: 1474–1483

Wakeling AE, Dukes M and Bowler J (1991) A potent specific pure antiestrogen with clinical potential. *Cancer Res* **51**: 3867–3873

Wakeling AE (1993) Are breast tumours resistant to tamoxifen also resistant to pure antioestrogens? *J Steroid Biochem Mol Biol* **47**: 107–111

 © Cancer Research Campaign 1999
